# Chlorogenic Acid Improves NAFLD by Regulating gut Microbiota and GLP-1

**DOI:** 10.3389/fphar.2021.693048

**Published:** 2021-06-30

**Authors:** Ameng Shi, Ting Li, Ying Zheng, Yahua Song, Haitao Wang, Na Wang, Lei Dong, Haitao Shi

**Affiliations:** ^1^ Department of Ultrasound, The Second Affiliated Hospital of Xi’an Jiaotong University, Xi’an, China; ^2^ Department of Geriatric Respiratory and Endocrinology (The Third Unit of Cadre’s Ward), The Second Affiliated Hospital of Xian Jiaotong University, Xi’an, China; ^3^ Department of Gastroenterology, The Second Affiliated Hospital of Xi’an Jiaotong University, Xi’an, China; ^4^ Department of Pharmacy, The Second Affiliated Hospital of Xi’an Jiaotong University, Xi’an, China

**Keywords:** chlorogenic acid, non-alcoholic fatty liver disease, gut microbiota, glucagon-like peptide-1, anti-inflammatory

## Abstract

Our previous studies have shown that chlorogenic acid (CGA) could significantly improve acute and chronic liver injury through antioxidant and anti-inflammatory activities. However, its effect on non-alcoholic fatty liver disease (NAFLD) are not entirely clear. This study aims to explore the effect of CGA on NAFLD induced by high-fat diet (HFD) and whether it regulates the gut microbiota and Glucagon-like peptide-1 (GLP-1). NAFLD mice were established by HFD and treated with or without CGA. Serum transaminase, fasting blood glucose (FBG), blood lipids, insulin, GLP-1 and lipopolysaccharide (LPS) were detected. Liver histology was evaluated with Hematoxylin-eosin staining. Toll like receptor 4 (TLR4) signaling pathway was analyzed with western blot and inflammatory cytokines were detected with real-time PCR. The content of gut microbiota were determined with real-time PCR of the bacterial 16S rRNA gene. Expressions of intestine tight junctional protein were examined with immunohistochemistry. CGA could alleviate HFD-induced hepatic steatosis and inflammation, reduce serum transaminase, FBG and blood lipids, increase insulin sensitivity. CGA also could reverse HFD-induced activation of TLR4 signaling pathway and expression of tumor necrosis factor-α (TNF-α) and interleukin-6 (IL-6) in liver. Meanwhile, CGA increased the content of Bifidobacterium and reduced the content of *Escherichia coli* in feces. Furthermore, CGA could increase the expression of tight junction proteins Occludin and zonula occludens-1 (ZO-1) in intestinal tissue. Moreover, CGA could the level of LPS and increased the level of GLP-1 in portal vein. These results indicated that CGA protected against HFD-induced hepatic steatosis and inflammation probably through its anti-inflammatory effects associated with regulation of gut microbiota and an increase of GLP-1 secretion and thus could be used as a potential drug for prevention and treatment of NAFLD.

## Introduction

Non-alcoholic fatty liver disease (NAFLD) has become the world’s most common chronic liver disease and is the main cause of end-stage liver disease (Mikolaevi et al., 2021). It is generally believed that NAFLD is associated with abnormal glucose and lipid metabolism, insulin resistance, inflammation, oxidative stress and imbalanced gut microbiota. To date, no evidence-based drug has been approved for the treatment of NAFLD (Campbell et al., 2021). Thus, revealing the pathogenesis of NAFLD and finding more effective therapeutic targets and drugs are challenges in the field of fatty liver research.

Gut *microbiota* imbalance participates in the occurrence and development of NAFLD (Aron-Wisnewsky et al., 2020). The structure of the gut microbiota in NAFLD patients is significantly different from that in the healthy population, which manifests as a reduction in beneficial bacteria (such as *Bifidobacterium* and *Lactobacillus*) and an increase in harmful bacteria (such as *Escherichia coli* and *Enterococcus*) (Dai et al., 2020). Overgrowth of the gut *microbiota* promotes the development of obesity and insulin resistance, which can damage the intestinal mucosa barrier function and allow endotoxins to travel to the liver, where they can induce or exacerbate liver inflammation and oxidative stress damage, leading to occurrence and progression of hepatic steatosis, inflammation and fibrosis (Akash et al., 2019).

Glucagon-like peptide-1 (GLP-1) is an important incretin and its receptor GLP-1R is widely distributed in tissues such as liver, pancreas, brain, heart and intestine. GLP-1 can increase liver fatty acid oxidation and insulin sensitivity by binding to GLP-1R, thereby improving NAFLD (Areti et al., 2020). Short-chain fatty acids (SCFA), a metabolic product of the gut *microbiota*, can regulate the secretion of GLP-1 (Bayer et al., 2018). Therefore, regulating gut *microbiota* and increasing the secretion of GLP-1 have become a new research direction for the treatment of NAFLD. Many studies have investigated the mechanism and feasibility of this therapeutic approach (Akash et al., 2019).

Chlorogenic acid (CGA) is a phenolic compound that is abundant in plants such as coffee, *eucommia ulmoides* and *honeysuckle*. CGA has antioxidative, anti-inflammatory, antitumor, antihypertensive and lipid-lowering functions (Shi et al., 2013). Our previous studies have shown that CGA can significantly improve carbon tetrachloride-induced acute liver injury and chronic liver injury through antioxidant and anti-inflammatory activities (Shi et al., 2013; Dong et al., 2016; Shi et al., 2017). It is also reported that CGA might also increase insulin sensitivity by increasing the secretion of GLP-1 (Mccarty, 2005; Faraji, 2019), however, the mechanism is not entirely clear. This study aims to explore the effect of CGA on NAFLD induced by HFD and whether it regulates the gut microbiota and Glucagon-like peptide-1 (GLP-1).

## Materials and Methods

### Reagents

CGA (#C3878) was purchased from Sigma-Aldrich (St. Louis, United States ). CGA was resolved in dimethyl sulfoxide and then sterile filtered before used and dissolved in distilled water. Rabbit-anti-mouse polyclonal primary antibodies against Toll like receptor 4 (TLR4), nuclear transcription factor-κB (NF-κB), p-NF-κB, inhibitor of NF-κB-α (IκB-α), *p*-IκB-α, β-actin, zonula occludens-1 (ZO-1), and Occludinwere purchased from Proteintech group. Goat-anti-rabbit secondary antibodies were purchased from Cell Signaling Technology.

### Experimental Animals

6-week-old male C57BL/6 mice (19.48 ± 2.32 g) were supplied by the Laboratory Animal Center of Xi’an Jiaotong University (Xi’an, China) and housed under specific pathogen-free conditions with free access to water. 24 mice were randomly divided into four groups (*n* = 6): Control:fed with a standard chow diet and treated with the same amount of distilled water; CGA:fed with a standard chow diet and treated with CGA (60 mg/kg, orally once a day); high-fat diet (HFD):fed with a high-fat diet and treated with the same amount of distilled water; HFD + CGA:fed with a high-fat diet and treated with CGA (60 mg/kg, orally once a day). HFD consists of 50% regular diet, 10% lard, 7.5% sucrose, 5% milk powder, 2.5% egg yolk powder, 15% soybean powder, and 10 drops of cod liver oil/100 g, which was similar to our previous approach (Li et al., 2019). The dosage and administration of CGA were chosen according our previous studies (Shi et al., 2013; Shi et al., 2017). After 12 w of feeding and 12 h of fasting, all mice were sacrificed by an intravenous administration of pentobarbital (1%, 50 mg/kg). Liver tissue, intestinal tissue at the ileum and *caecum*, portal vein serum and stool specimens were collected using sterilizing tubes after 12 w feeding and were immediately frozen in liquid nitrogen and stored at −80°C.

### Blood Analysis and Enzyme-linked Immunosorbent Assay

Serum transaminase, fasting blood glucose (FBG) and blood lipids including total cholesterol (TC) and triglyceride (TG) were analyzed by a biochemistry analyzer (Olympus AU2700, Japan). Sandwich Enzyme-linked immunosorbent assay (ELISA) (R and D Systems, Minneapolis, MN, United States ) was used to analyze the content of fasting insulin (FINS), lipopolysaccharide (LPS and GLP-1) according to the manufacturer’s instructions. The homeostasis model assessment-estimated insulin resistance (HOMA-IR) index was calculated as fasting plasma glucose level (mmol/L) × fasting insulin level (mIU/L)/22.5 (Matthews et al., 1985). ELISA assay protocol: after placed at 4°C overnight, whole blood samples were centrifuged at 1,000 g for 20 min to prepare serum. 100°μl of standard or sample were added to the standard hole and sample hole in ELISA plate, no reagent added to blank hole. After fully mixed, the reaction plate was incubated at 37°C for 120°min; the reaction plate was washed 4–6 times; 100 μl of the first antibody working solution was added and incubated at 37°C for 60°min; after washed, 100 μl of enzyme labeled antibody working solution was added and incubated at 37°C for 30°min; after washed, 100 μl of substrate working solution was added and reacted at 37°C in dark for 15°min; 100 μl termination solution were added and the absorbance were measured at 450 nm within 30 min. The standard curve was made and the content of target protein (FINS, LPS and GLP-1) was calculated.

### Hematoxylin-Eosin Staining

Liver tissues fixed in 10% formaldehyde were cleared in xylene, dehydrated with gradient ethanol, embedded in paraffin, and serially sectioned at 5 μm. Sections were dewaxed with xylene, dehydrated with gradient ethanol, stained with Harris’s hematoxylin for 5 min, differentiated with 0.1% hydrochloric acid alcohol, stained with 1% eosin for 2 min, dehydrated by gradient ethanol, cleared in xylene, mounted with neutral gum, and observed and photographed under a microscope. NAFLD Activity Score (NAS) defined as the sum of steatosis, lobular inflammation and ballooning was used to evaluate improvement in liver histology (Kleiner et al., 2005).

### Western Blot

The total protein of liver tissues stored at 80°C was extracted using RIPA protein lysis buffer (Beyotime, Shanghai, China). Samples of 50 μg of protein were mixed with gelloading buffer, boiled for 5 min, and loaded on 10% polyacrylamide gels. After electrophoresis, the proteins were transferred to PVDF membranes (Millipore, Billerica, MA, United States ). Non-specific antibody binding was blocked by preincubation of the membranes in 1× Tris-buffered saline (TBS) containing 5% skimmed milk for 2 h at room temperature. The membranes were incubated overnight at 4°C with rabbit-anti-mouse primary antibodies TLR4 (1:500), IκB-α(1:500), p-IκB-α(1:500), NF-κB (1:400), p-NF-κB (1:400) and β-actin (1:1,000) in 1 × TBS containing 5% skimmed milk. After washing, they were incubated for 2 h at room temperature with goat-anti-rabbit secondary antibody at a 1:2,000 dilution. Positive bands were detected using an ECL plus chemiluminescence detection kit (Millipore, Billerica, MA, United States ). The results were analyzed using Gel-pro Analyzer 4.0 software (Media Cybernetics, CA, United States ), normalized to β-actin.

### Real-Time PCR

The mRNA expression of TNF-α and IL-6 in liver tissues were detected with real-time PCR. Total RNA from liver tissues was extracted using a TRIzol kit (Invitrogen, Los Angeles, CA, United States ) according to the manufacturer’s protocol and synthesized into cDNA using a RevertAid™ First Strand cDNA Synthesis Kit (Fermentas, Thermo Scientific Molecular Biology, Waltham, MA, United States ). The primer sequences of TNF-α and IL-6 are shown in Table 1. The real-time PCR was performed on an ABI 7,500 Real-Time Detection System using the SYBR Premix Ex Taq II qRT-PCR Kit (TaKaRa) to obtain the cycle threshold (CT) values. The applied PCR conditions were: preliminary denaturation at 95°C for 30°s, followed by 40 cycles at 95°C for 5°s and 60°C for 30°s. The relative expression levels of target genes were calculated using the 2^−ΔΔCT^ method (Livak and Schmittgen, 2002), β-actin was used as an internal control.

**TABLE 1:** Primer sequences used for gut microbiota and inflammatory cytokines.

Microbiota/genes	Sequences
*Bifidobacterium*	Forward: 5′-GGG​TGG​TAA​TGC​CGG​ATG-3′
	Reverse: 5′-TAA​GCG​ATG​GAC​TTT​CAC​ACC-3′
*Lactobacillus*	Forward: 5′-AGC​AGT​AGG​GAA​TCT​TCC​A-3′
	Reverse: 5′-CAC​CGC​TAC​ACA​TGG​AG-3′
*Escherichia coli*	Forward: 5′-GTT​AAT​ACC​TTT​GCT​CAT​TGA-3′
	Reverse: 5′-ACC​AGG​GTA​TCT​AAT​CCT​GTT-3′
*Enterococcus*	Forward: 5′-CCC​TTA​TTG​TTA​GTT​GCC​ATC​ATT-3′
	Reverse: 5′-ACT​CGT​TGT​ACT​TCC​CAT​TGT-3′
TNF-α	Forward: 5′-GCA​TGA​TCC​GCG​ACG​TGG​AA-3′
	Reverse: 5′-AGA​TCC​ATG​CCG​TTG​GCC​AG-3′
IL-6	Forward: 5′-ACC​CCA​ATT​TCC​AAT​GCT​CTC-3′
	Reverse: 5′-AAC​GCA​CTA​GGT​TTG​CCG​AG-3′
β-actin	Forward: 5′-GGC​TGT​ATT​CCC​CTC​CAT​CG-3′
	Reverse: 5′-CCA​GTT​GGT​AAC​AAT​GCC​ATG​T-3′

The stool counts of *Bifidobacterium*, *Lactobacillus*, *Escherichia coli* and *Enterococcus* were determined from real-time PCR of the bacterial 16S rRNA gene. Total DNA was isolated from the fecal samples using a DNeasy Blood and Tissue Minikit (Qiagen, Germany) accroding to the manufacturer’s protocols. The primer sequences of Bifidobacterium, Lactobacillus, *Escherichia coli* and *Enterococcus* used are shown in Table 1. The real-time PCR was performed similar to the above. CT values and standard curve were obtained by amplifying 10-fold serially diluted plasmids. Each amplification efficiency was also determined. The number of *Bifidobacterium*, *Lactobacillus*, *Escherichia coli* and *Enterococcus* copies were calculated based on their standard curves, respectively.

### Immunohistochemistry

Liver and intestine tissues fixed in formalin and then embedded in paraffin were cut into 5-μm sections. After deparaffinized and rehydrated, the sections were antigen retrieved in the sodium citrate buffer solution and serum blocked. Then the sections were incubated with primary antibodies at 4°C overnight. The applied primary antibody: rabbit-anti-mouse polyclonal against ZO-1 (1:100) and Occludin (1:100). After washing with phosphate-buffered saline (PBS) the next day, biotin-labeled secondary antibody (1:500) and streptavidin-biotin-peroxidase were added dropwise, followed by 3,3′-diaminobenzidine staining, counterstaining by hematoxylin, dehydration by gradient alcohol, clearing in xylene, and mounting with neutral gum. The primary antibody was replaced by PBS as a negative control. The slides were scanned at 100 × under light microscope (Olympus, Tokyo, Japan). Five fields were randomly selected for each slide. Image-Pro Plus 6.0 software (Media Cybernetics, Maryland United States ) was used to calculate the ratio of the positively stained area to the area of the field of view, and the mean density from the five fields was taken.

### Statistical Analysis

SPSS 17.0 was used for statistical analysis. All data showed normal distribution and are expressed as the mean ± standard deviation. Comparisons between multiple groups were performed using one-way analysis of variance (ANOVA). The LSD-*t* test was used for comparisons between groups. *p* < 0.05 was considered statistically significant.

## Results

### Effect of Chlorogenic Acid on the Liver Pathology

**FIGURE 1:**
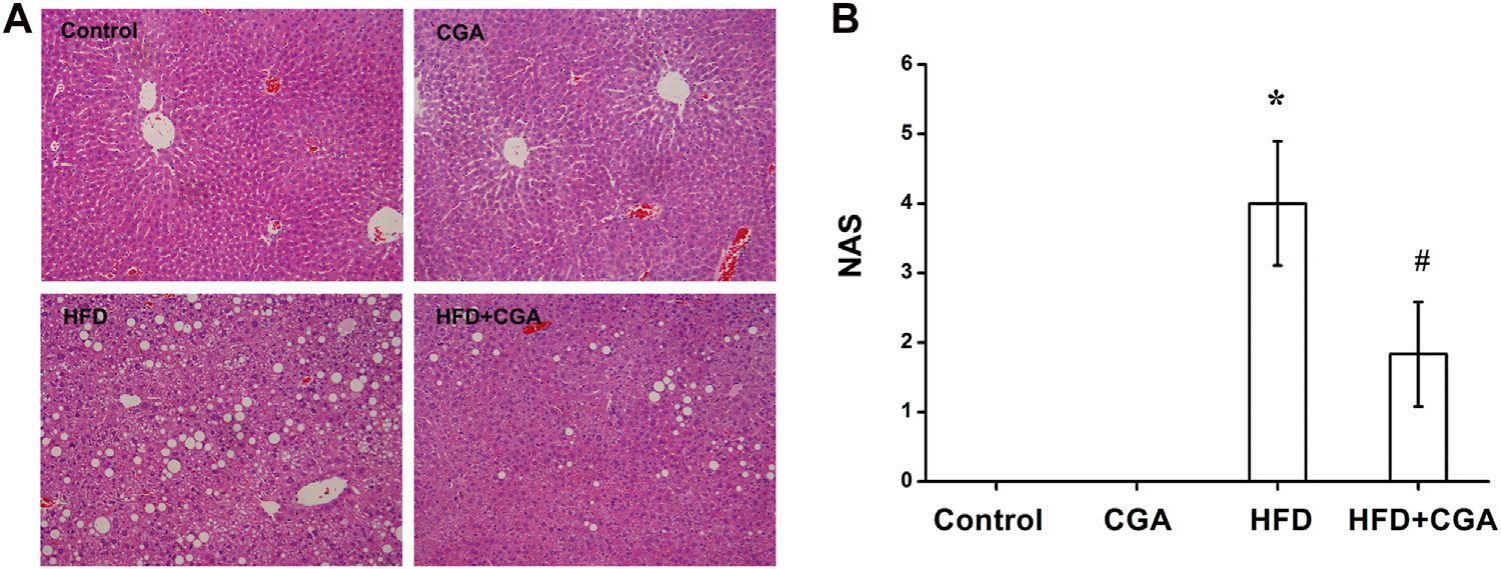
Effect of CGA on liver tissue pathology. **(A)** Hematoxylin-eosin staining for liver sections (200×). **(B)** NAS in four groups. Data are presented as the mean ± SD. ^*^ *p* < 0.05 as compared with the control group; ^#^ *p* < 0.05 as compared with the HFD group.

In the negative control group and CGA control group, the liver cells were arranged neatly, and the morphology of the hepatic lobule was regular. In the HFD group, the liver cells were disorderly and showed obvious steatosis, mainly with microbubble-like lipid droplets, accompanied by a large amount of inflammatory cell infiltration and patchy necrosis. The degree of hepatic steatosis was lesser in the CGA treatment group than the HFD group, and there were significantly fewer inflammatory cells (Figure 1A). NAS defined as the sum of steatosis, lobular inflammation and ballooning was used to evaluate hepatic steatosis and inflammation. Compared with HFD group, the NAS score was significantly lower in HFD + CGA group (*p* < 0.05) (Figure 1B).

### Effect of Chlorogenic Acid on Serum Transaminase, Fasting Blood Glucose, Blood Lipids and Insulin Resistance

**FIGURE 2:**
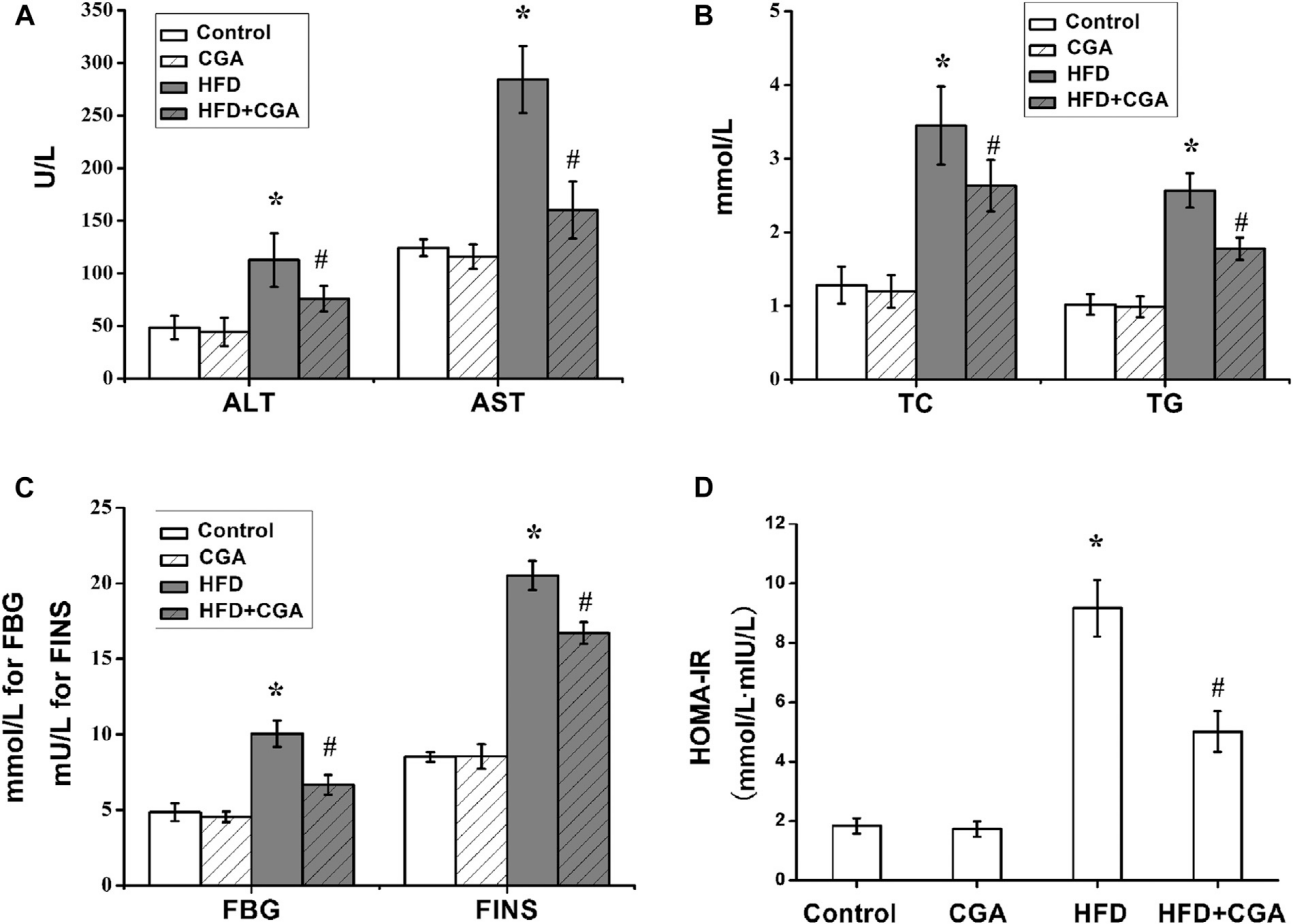
Effect of CGA on serum transaminase, fasting blood glucose (FBG) and blood lipids and insulin resistance. **(A)** serum transaminase; **(B)** blood lipids including total cholesterol (TC) and triglyceride (TG); **(C)** FBGand FINS; **(D)** HOMA-IR. Data are presented as mean ± SD. ^*^ *p* < 0.05 as compared with the control group; ^#^ *p* < 0.05 as compared with the HFD group.

To evaluate the effect of CGA on metabolic characteristics of NAFLD mice, serum transaminase, FBG, blood lipids and fasting insulin were detected. Serum transaminase, FBG, blood lipids and HOMA-IR of the control group and CGA control group were in the normal range. The mice in the HFD group showed significantly elevated serum transaminase, FBG, blood lipids and HOMA-IR (*p* < 0.05). After treatment with CGA, the serum transaminase, FBG, blood lipids and HOMA-IR significantly decreased (*p* < 0.05) (Figure 2).

### Effect of Chlorogenic Acid on Liver Inflammation

**FIGURE 3:**
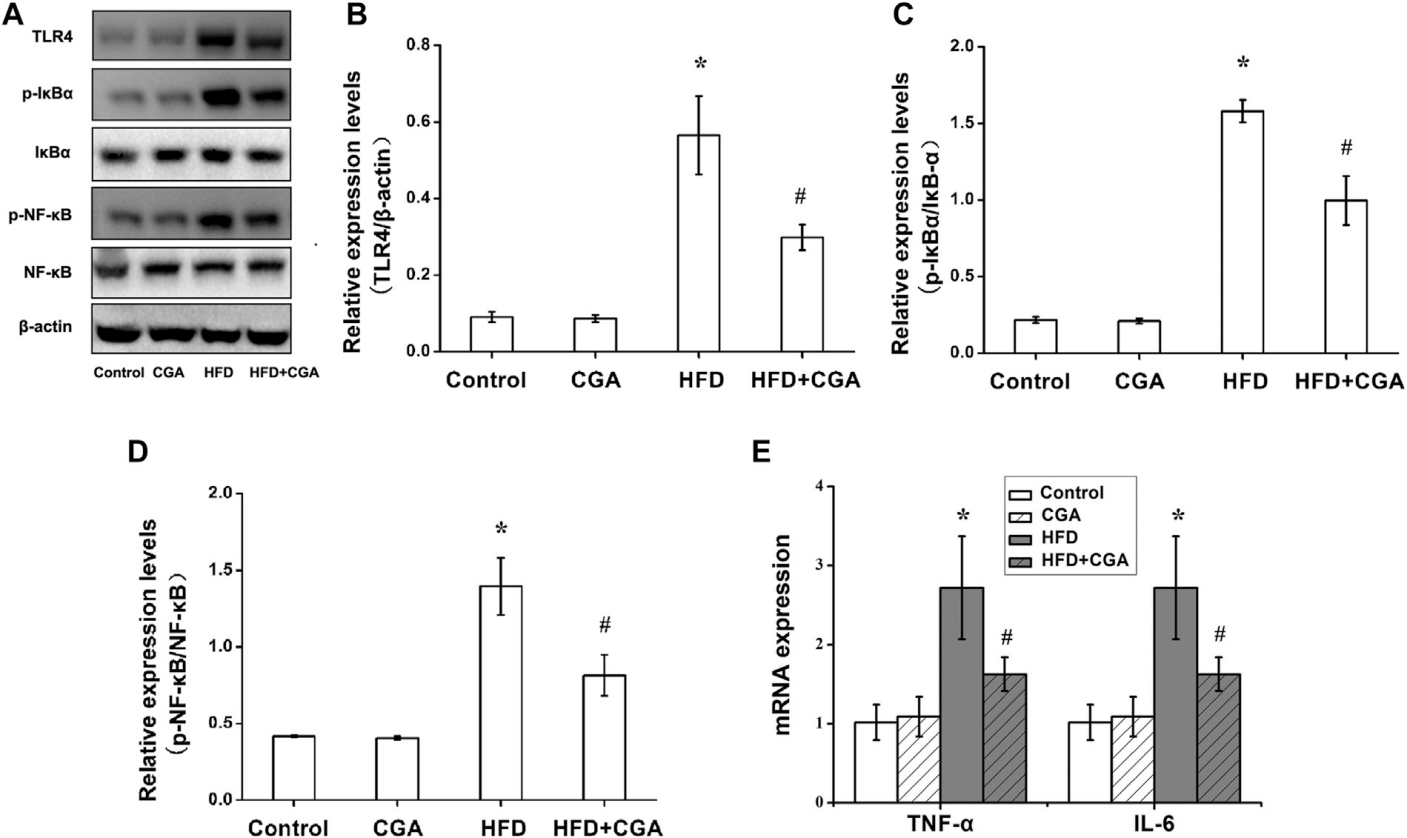
Effects of CGA on the TLR4/NF-κB signaling pathway and inflammatory cytokines in the liver. **(A)** TLR4/NF-κB signaling pathway analyzed with western blot. **(B)** TLR4 expression. **(C)** *p*-IκBα/IκBα expression **(D)** p-NF-kB/NF-kB expression. **(E)** expression of TNF-α and IL-6 analyzed with real-time PCR. Data are presented as mean ± SD. ^*^ *p* < 0.05 as compared with the control group; ^#^ *p* < 0.05 as compared with the HFD group.

TLR4 signaling pathway is an important pathway related to liver inflammation in NAFLD. TNF-α and IL-6 were two major inflammatory cytokines. We further evaluated the effect of CGA on liver inflammation in NAFLD. Compared with the control group, the HFD group had significantly higher protein expression of TLR4, p-NF-κB, and *p*-IκB-α in the liver (*p* < 0.05), and the mRNA expression levels of TNF-α and IL-6 were significantly increased (*p* < 0.05). After CGA treatment, the protein expression of TLR4, p-NF-κB, and *p*-IκB-α and mRNA expression of TNF-α and IL-6 were all decreased (*p* < 0.05). However, the CGA itself had no significant effect on the expression of TLR4 signaling pathway and inflammatory cytokines (Figure 3).

### Effect of Chlorogenic Acid on Gut Microbiota

In order to elucidate the mechanism of CGA improving liver inflammation, we examined the effect of CGA on gut-liver axis, and the gut microbiota was detected. Compared with the control group, the HFD group had more *Escherichia coli* and *Enterococcus* and less *Bifidobacterium* and *Lactobacillus* (*p* < 0.05). Treatment with CGA increased the content of Bifidobacterium, reduced the content of *Escherichia coli* (*p* < 0.05). However, CGA had no significant effect on the content of *Lactobacillus* and *Enterococcus* (Table 2).

**TABLE 2:** Quantitation analysis of gut microbiota in fecal samples by real-time PCR (Mean ± SD).

Group	*Bifidobacterium*	*Lactobacillus*	*Escherichia coli*	*Enterococcus*
Control	8.45 ± 0.29	6.39 ± 0.33	8.44 ± 0.29	7.44 ± 0.34
CGA	8.62 ± 0.31^a^	6.41 ± 0.19	8.09 ± 0.13^a^	7.39 ± 0.33
HFD	8.05 ± 0.38^a^	5.83 ± 0.39^a^	8.71 ± 0.26^a^	8.46 ± 0.15^a^
HFD + CGA	8.32 ± 0.26^b^	5.85 ± 0.23	8.13 ± 0.36^b^	8.45 ± 0.31

Data were expressed as log_10_ (copy/g feces).

a *p* < 0.05 as compared with the control group.

b *p* < 0.05 as compared with the HFD group.

### Effect of Chlorogenic Acid on the Expression of Intestine Tight Junction Proteins

**FIGURE 4:**
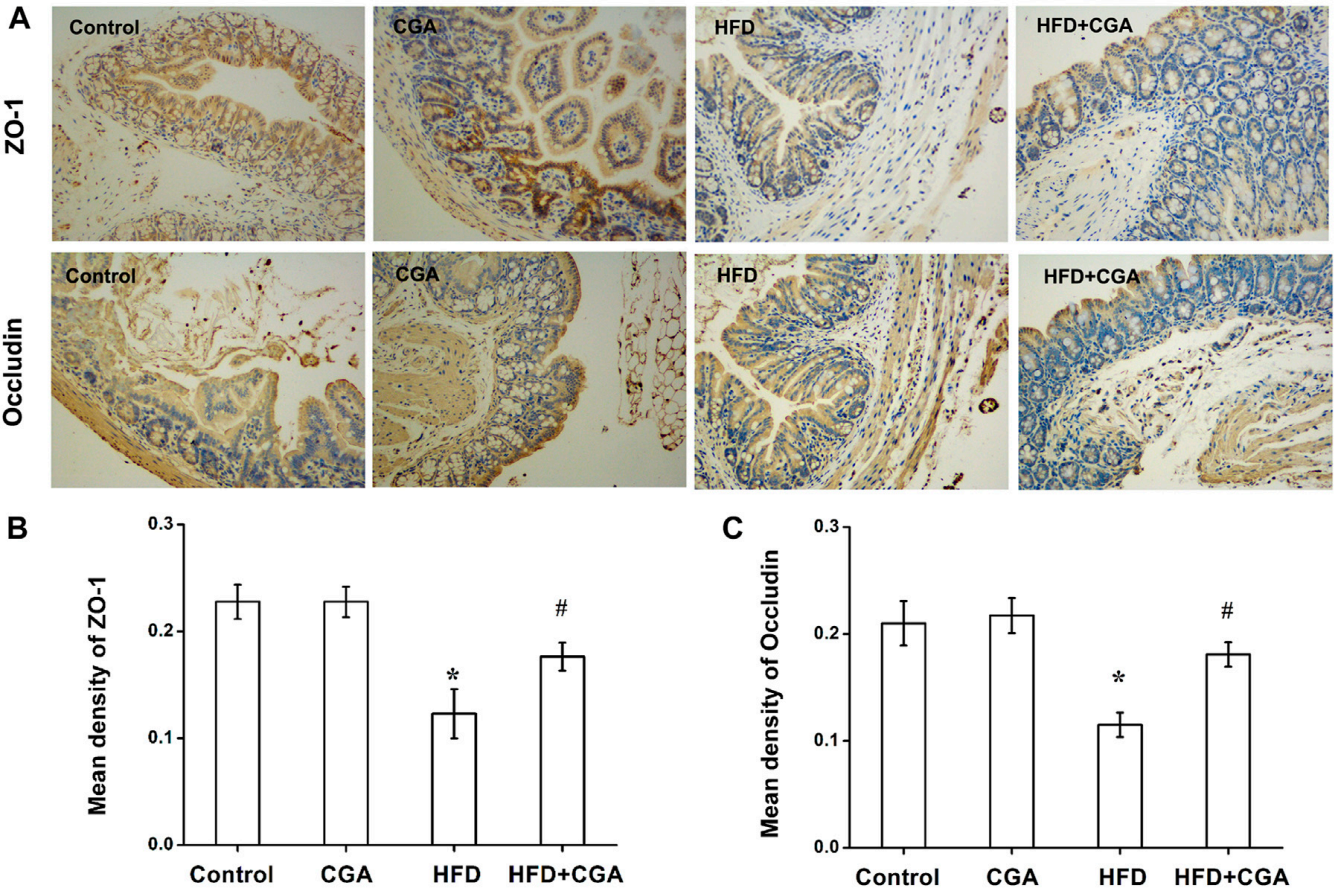
Effect of CGA on the expression of intestinal tight junction protein. **(A)** Immunohistochemical staining of ZO-1 and Occludin (100×) **(B)** Mean density of ZO-1 analyzed with Image-Pro Plus. **(C)** Mean density of Occludin analyzed with Image-Pro Plus. Data are presented as mean ± SD. ^*^ *p* < 0.05 as compared with the control group; ^#^ *p* < 0.05 as compared with the HFD group.

The gut microbiota plays an important role in integrity maintenance of intestinal mucosal barrier and intestinal tight junction protein is the principal determinant of mucosal barrier. Compared with the control group, the expression of the tight junction protein Occludin and ZO-1 in the HFD group were downregulated (*p* < 0.05), and the expression of Occludin and ZO-1 were increased after CGA treatment (*p* < 0.05). However, the CGA itself had no significant effect on the expression of the tight junction proteins (Figure 4).

### Effect of Chlorogenic Acid on the Level of Lipopolysaccharide in the Portal Vein

**FIGURE 5:**
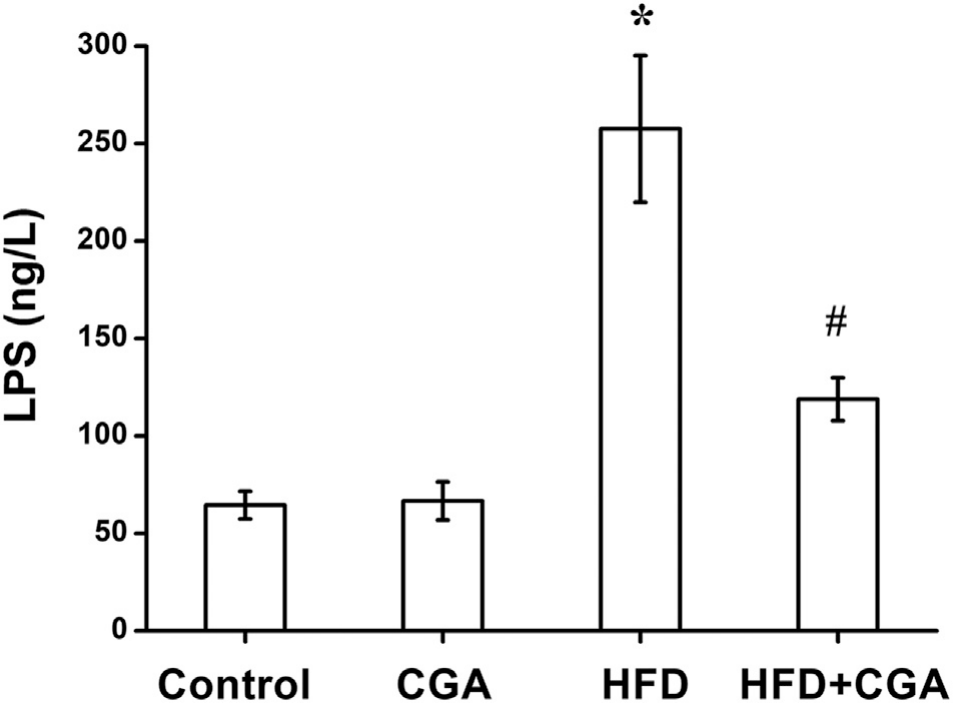
Effect of CGA on the level of LPS in portal vein. Data are presented as mean ± SD. ^*^ *p* < 0.05 as compared with the control group; ^#^ *p* < 0.05 as compared with the HFD group.

Disruption of gut microbiota and intestinal mucosal barrier leads to the increase of mucosal permeability and the entry of endotoxin into the liver. Therefore, we examined the level of LPS in portal vein. Compared with the control group, the level of LPS in portal vein significantly in the HFD group was increased (*p* < 0.05), and the level of LPS was reduced after CGA treatment (*p* < 0.05). However, CGA itself had no significant effect on the level of LPS (Figure 5).

### Effect of Chlorogenic Acid on the Level of GLP-1 in the Portal Vein

**FIGURE 6:**
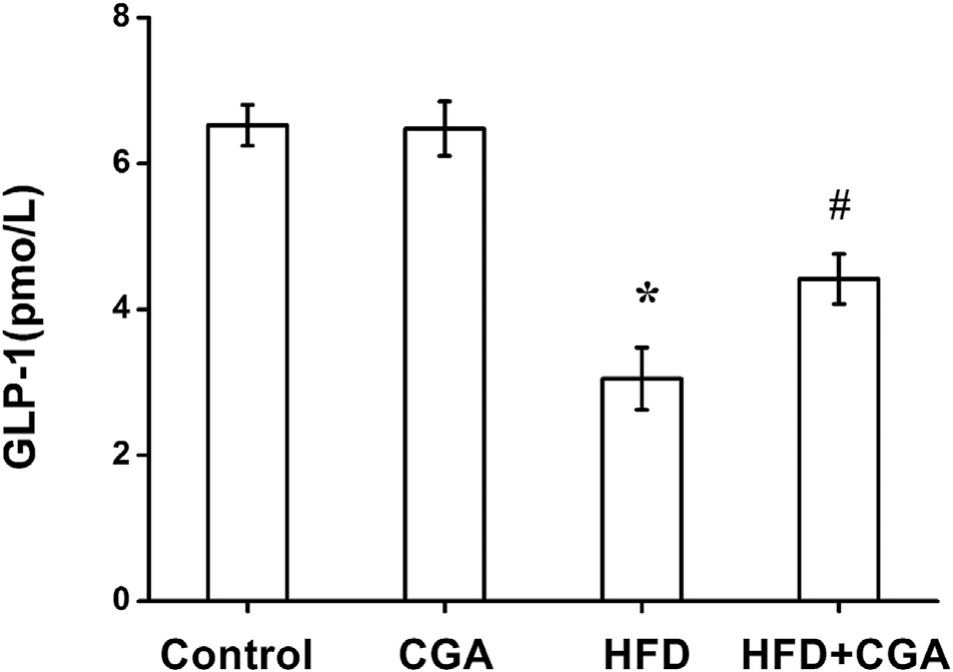
Effect of CGA on the level of GLP-1 in portal vein. Data are presented as mean ± SD. ^*^ *p* < 0.05 as compared with the control group; ^#^ *p* < 0.05 as compared with the HFD group.

Gut microbiota and its metabolites can increase the secretion of GLP-1, which thus improve insulin resistance, hepatic steatosis and inflammation. Compared with the control group, the level of GLP-1 in portal vein significantly in the HFD group was decreased (*p* < 0.05), and the level of GLP-1 was increased after CGA treatment (*p* < 0.05). However, CGA itself had no significant effect on the level of LPS (Figure 6).

## Discussion

NAFLD seriously endangers people’s health when it develops into fatty hepatitis, liver cirrhosis and even liver cancer. In recent years, the influence of gut microbiota as a “microbial organ” on human health has received extensive attention. Gut microbiota disturbances can be a manifestation of certain diseases and can also cause or exacerbate diseases, such as obesity, diabetes mellitus, metabolic syndrome, inflammatory bowel disease and tumors (Illiano et al., 2020). Gut microbiota dysregulation plays an important role in the occurrence and progression of NAFLD (Dai et al., 2020). Overgrowth of the gut microbiota leads to dysfunction of the intestinal mucosal barrier, resulting in the translocation of endotoxins to the liver, where they bind to their specific receptors CD14 and TLR4 on Kupffer cells. They release a series of oxygen free radicals and inflammatory cytokines by activating downstream signaling pathways such as NF-κB to cause changes in the liver microenvironment, which induces or aggravates inflammation and oxidative stress damage in the liver (Hussain et al., 2020). Therefore, the gut microbiota, intestinal mucosal barrier, endotoxins and NAFLD are interrelated.

Incretins are a class of intestinal hormones in the human body, including GLP-1 and glucose-dependent insulinotropic peptide (GIP). GLP-1 is a product of the translation and posttranslational processing of the proglucagon gene. It is secreted by L cells of the small intestine and the large intestine and is rapidly degraded by dipeptidyl peptidase 4 (DPP-4) (Brierley et al., 2021). By binding to GLP-1R, GLP-l regulates not only glucose metabolism but also lipid metabolism (Karstoft et al., 2015; Lutz and Osto, 2016). An abnormal incretin system is present in NAFLD patients. It is found that nondiabetic NAFLD and non-alcoholic steatohepatitis (NASH) patients had lower GLP-1 than healthy controls (Christine et al., 2014). The mechanisms by which GLP-1 improves NAFLD include direct and indirect effects (Teshome et al., 2020). GLP can activate AMP-activated protein kinase (AMPK) to increase liver lipid oxidation, improve insulin sensitivity and inhibit liver fat synthesis. GLP-1 also regulates feeding neurotransmitters through central GLP-1R, leading to anorexia and reduced food intake, leaving less material for liver fat synthesis. Through gastric GLP-1R, GLP-1 inhibits gastric emptying and improves satiety, thereby indirectly reducing food intake.

There is clear evidence that the gut microbiota is related to the incretin effect, the gut microbiota does affect the differentiation and apoptosis of intestinal epithelial cells (Demidova et al., 2020). Adding prebiotics to the diet can increase the amount of bifidobacterial in the distal gut, increase the fermentation of dietary fiber, promote the differentiation of colonic L cells, and increase the secretion of GLP-1. Short-chain fatty acids, among the metabolites of gut microbiota, can regulate GLP-1 secretion, thereby improving diet-induced obesity and insulin resistance (Jiao et al., 2020). This study shows that CGA could increase the contents of *Bifidobacterium* and reduced the *Escherichia coli* in NAFLD mice, suggesting its promoting effect on GLP-1 secretion may be related with its effect on these gut microbiota. Some studies have shown that CGA could increase the diversity of gut microbiota (Song et al., 2019; Yan et al., 2020), while others have shown that the CGA treated mice showed similar microbial structure compared to that of the HFD mice (He et al., 2020). Based on these researches and our study, we speculated that CGA could regulate composition of gut microbiota under different pathological conditions as a different way. There is a certain negative correlation between coffee consumption and the occurrence and progression of chronic liver diseases, including fatty liver and liver fibrosis. Some epidemiological studies found that the risk of NAFLD in patients who drank coffee was significantly lower than that in patients who did not and a significantly decreased risk of liver fibrosis among NAFLD patients who drank coffee (Bambha et al., 2015; Wijarnpreecha et al., 2017; Calabro et al., 2020). Vitaglione et al.found that coffee supplementation prevented HFD-induced NAFLD in mice by reducing hepatic fat deposition and modulating pathways of the gut-liver axis and gut microbiota (Vitaglione et al., 2019). It remains unclear which ingredients of coffee play these roles. One recent study showed that caffeic acid, one compound of coffee, prevents non-alcoholic fatty liver disease induced by a high-fat diet through reverting the imbalance in the gut microbiota and related lipopolysaccharide-mediated inflammation (Hnm et al., 2021). CGA is a polyphenolic compound found in high amounts in coffee. CGA has significant antioxidant, anti-inflammatory, lipid-lowering, and hypoglycemic effects. Our and other’s studies showed that CGA can improve acute and chronic liver injury caused by various reasons (Dong et al., 2016; Shi et al., 2017; Kim et al., 2018; Abbas et al., 2020; Hu et al., 2020). Chlorogenic acid can also improve NAFLD through its antioxidant, anti-inflammatory, lipid-lowering activities (Alqarni et al., 2019; Zamani-Garmsiri et al., 2020). However, there are few studies on the effect of CGA on gut microbiota, intestinal mucosal barrier, short-chain fatty acids and GLP-1 in NAFLD patients or animal models. Xie M found CGA can alleviate colitis in HFD-induced obesity through its anti-inflammatory effects associated with changes in gut microbiota composition and an increase in the production of short-chain fatty acids (Xie et al., 2021). Mccarty MF found that CGA could increase GLP-1 secretion, thereby reducing the risk of diabetes (Mccarty, 2005). This study showed that CGA significantly improved liver damage, reduced FBG and blood lipids, improved insulin resistance, and reduced hepatic steatosis and inflammation in NAFLD mice. We also found that CGA regulated the gut microbiota, improved intestine mucosal barrier, reduced LPS levels, and increased GLP-1, suggesting that CGA could improve hepatic steatosis and inflammation by enhanceing the proportions of beneficial bacteria to increase the level of GLP-1. In the research method, the mice received CGA and HFD at the same time, so our results could better explain the potential protective and preventive effects of CGA in the development of NAFLD. If we gave CGA intervention after NAFLD model was established, it could better explain its therapeutic effect. This needs to be further studied in the future.

## Conclusion

In summary, these results indicate that CGA protects against HFD-induced hepatic steatosis and inflammation probably through its anti-inflammatory effects associated with regulation of certain gut microbiota and an increase of GLP-1 secretion and thus can be used as a potential drug for the prevention and treatment of NAFLD. Meanwhile, this study also elucidated the new mechanism of anti-inflammatory effect of CGA from gut-liver axis. However, the mechanism of CGA regulating gut microbiota and GLP-1 needs further study.

## Data Availability

The datasets presented in this study can be found in online repositories. The names of the repository/repositories and accession number(s) can be found in the article/Supplementary Material.
